# Pharmaceutical marketing strategies’ influence on physicians' prescribing pattern in Lebanon: ethics, gifts, and samples

**DOI:** 10.1186/s12913-019-3887-6

**Published:** 2019-01-30

**Authors:** Micheline Khazzaka

**Affiliations:** Independent Researcher, Zahle, Lebanon

**Keywords:** Pharmaceutical marketing, Prescribing behavior, Ethics, Physicians’ profile

## Abstract

**Background:**

Drug companies rely on their marketing activities to influence physicians. Previous studies showed that pharmaceutical companies succeeded to manage physicians prescribing behavior in developed countries. However, very little studies investigated the impact of pharmaceutical marketing strategies on prescribing pattern in developing countries, middle-eastern countries. The objective of this research was to examine the influence of drug companies’ strategies on physicians’ prescription behavior in the Lebanese market concerning physicians’ demographic variables quantitatively. Moreover, this study tested whether Lebanese physicians considered gifts and samples acceptance as an ethical practice.

**Methods:**

Sampling was done by using a non-probability method. An online cross-sectional study was conducted through WhatsApp. A self-administered questionnaire survey was conducted during the months of February and March 2018. Cronbach’s Alpha reliability coefficient was calculated. Data were statistically analyzed by using IBM SPSS statistics version 24 software. Chi-square and Cramer’s v tests were used to finding sign correlation, and Spearman test was used to measure the strength and direction of a relationship between variables.

**Results:**

Results found that pharmaceutical marketing strategies are correlated to physicians’ prescribing behavior. We demonstrated that the majority of the promotional tools tested were mostly or sometimes motivating physicians to prescribe promoted drugs. The major tools that physicians agreed to be mostly motivated by are visits of medical representatives and drug samples while sales calls made by pharmaceutical companies are the less influential tool. Regarding gift acceptance, this study demonstrated that physicians consider gifts’ acceptance as a non-ethical practice. Results showed that most physicians use free samples to treat their patients.

We demonstrated that there is a relationship between physicians’ prescribing pattern and their age, gender and the location of practice.

**Conclusions:**

Findings of this study provided an insightful work, serving as one of the first humble steps in the imminent direction of merging this paper with the previous literature. From a managerial perspective, pharmaceutical marketing managers of drug companies can use the research findings to design better their strategies directed to the Lebanese physicians who can also benefit from the results obtained.

**Electronic supplementary material:**

The online version of this article (10.1186/s12913-019-3887-6) contains supplementary material, which is available to authorized users.

## Background

Pharmaceutical marketing efforts directed to physicians are getting more and more attention over the years. There are many tactics adopted by pharmaceutical companies [1] such as physicians-targeted promotions which are free samples, journal advertisements [2], printed product literature and other gifts that helped them to increase the acceptability of their products [3]. On average, pharmaceutical companies spent 20% or more of their sales on marketing [4] which made them a lot of money, and they had little incentive to stop those tactics [5]. It was estimated that 84% of pharmaceutical marketing efforts are directed toward physicians [2] because from the manufacturer’s point of view, physicians are the key decision makers [6–8], the gatekeepers to drug sales [9]. The structure of pharmaceutical markets differs from country to country because it has a national character. However, the pharmaceutical industry has an international nature [4]. To the best of our knowledge, few published studies addressed the situation in the developing world, and very few were those in the middle-eastern countries.

In Lebanon, inappropriate prescribing practices for certain prescription drugs are a common problem. One study found that 40% of all prescriptions in seven hospitals in Lebanon contained an error, of which 9% were unnecessary medication prescription [10]. One of the explanations to this observation might be because of physician-targeted promotions [2] adopted by pharmaceutical companies to increase the acceptability of their products [3].

Although many articles contemplated various theories of marketing influence on physicians, there is still a gap to fill. Ethical acceptability of gifts and samples is a comparatively new topic drawing momentous attention. Given the interdisciplinary nature of this research, it was appropriate for us to consider relevant theories from some different disciplines: diffusion and adoption theory, advertising theory, agency theory and role theory that influence the individual and the environment in which new products are adopted (drugs prescribed). Therefore, this study covered physicians’ opinion regarding the ethical acceptability of gifts and samples and highlighted physicians’ profile and demographic parameters influencing their prescribing behavior.

This desk research was an outcome of the authors’ academic and professional interest in the subject of pharmaceutical marketing and its impact on physicians’ prescribing pattern, especially in the mentioned country.

From an academic view, this present study aimed to add knowledge to the existing literature. It was an aggregate account of the connected exploration across disciplines with practical connotations.

Therefore, this investigation subtly attempted to bring substantial academic and managerial implications.

Academically, in searching existing literature, the present study identified some gaps. The majority of researches conducted were in developed countries. Developing countries received quite a bit of attention.

Therefore, a study of drug companies’ impact on Lebanese physicians prescribing behavior would report an original empirical research and would inform practice and future research by providing an insight into which extent pharmaceutical marketing strategies influenced Lebanese physicians prescribing behavior and what was the most influential strategy. No scholarly article merged in one study pharmaceutical marketing strategies in Lebanon, their impact on physicians prescribing pattern and physicians opinion regarding ethical acceptability of gifts and samples to find the correlation between them. This paper was virtually the first to advocate such a reform, and it served as one of the first humble steps in the imminent direction of merging this paper with the previous literature.

From a managerial perspective, a good understanding of drug companies influence on physicians provided pharmaceutical managers a framework to optimize promotion activities by firstly deciding where to focus their efforts to increase their benefits and secondly by choosing the best promotional approach and tool to persuade physicians best and thus avoiding any wasteful expenditure [8]. Additionally, the observance of ethical theories for medicinal drug promotion by doctors contributed to a more rational prescription of drugs. From another hand, when drug companies had more influence on some physicians and not on others, companies’ managers began to address the question regarding physicians opinion about the ethicality of receiving gifts and samples as a factor preventing these doctors from being influenced. If there was a difference in physicians’ opinion, managers then began to address possible reasons for the differences in answers observed such as demographic factors and physicians’ profile (location of practice, age or year of graduation and its influence on rates of adoption, gender, and nature of practice).

Conducting such a scholarly study from a managerial perspective aimed at highlighting functional implications and future research possibilities was of equal interest to academia and professionals.

The primary focus of the pharmaceutical industry was on profitability [11]. Thus, it was trying to leave a lasting impression on the prescribers (physicians) mind [12]. Normative principles of justice and fidelity required that physicians stay free from outside influence with regards to decisions about patient care [11].

Thus, it would be helpful for both, physicians and pharmaceutical industries to be aware of ethical theories and ethical principles [13]. The four fundamental ethical theories that are among the most frequently discussed in the business ethics literature are egoism [14], utilitarianism, deontology [15], and social justice.

### The application of ethical theories during interaction with the pharmaceutical industry

The purpose of these theories was to help doctors and pharmaceutical companies’ managers acquire insight into their beliefs about the many criticisms that were made against marketing and, especially, insight about where they stood on the morally difficult situations that confronted them and what actions they would take in response to them. In practical ethics, two concepts existed while making decisions: utilitarian and deontological. In utilitarian ethics, consequences justified the ways to achieve it, but in deontological ethics, duties were of significant importance and outcomes may not justify the means [13].

Pharmaceutical marketing personalized to physicians such as the provision of samples and gifts raised such ethical issues [16]. To make effective decisions, the key was to think about different choices regarding their ability to accomplish one of the physicians’ most important goals that were ethical prescribing of drugs. Five ethical principles were identified as the cornerstone of the ethical guidelines [17], they helped to explain and to clarify the issues involved in a specific dilemma, and they were globally valuable to approach ethical and appropriate decision-making: beneficence, nonmaleficence, respect for autonomy, justice, and fidelity [18].

### Marketing and promotion practices regarding the Lebanese code of ethics

In Lebanon, in 2016, the Lebanese Ministry of Public Health undertook the initiative to stipulate a Code of Ethics in partnership with the concerned parties to set regulatory frameworks. These frameworks ensured respect of legal, ethical, and scientific principles in the medicinal market as well as enhancing the rational use of drugs to prevent practices that do not comply with ethics by acting as a reliable reference to marketing practices. It was established in accordance with the Lebanese cultural context leading to the particular need for this research in Lebanon since no well-documented studies were done previously. The Lebanese Code of Ethics was divided into three main components which are: Marketing and Promotion Practices, Implementation Procedures, and the Pledge and signature [19].

While we were concerned with the process of prescription (adoption of drugs), we must also take into account many reasons why prescribing a specific medication may be unaccepted by some physicians and not by others [20, 21]. Variables such as the degree of socialization, proximity to peers, rural vs. urban local of practice, do exist between product message and the act of prescribing.

Research by Tamblyn, McLeod, Hanley, Girard, and Hurley (2003) [22] indicated that new drug utilization was lower among generalists and specialists practicing in rural. In consideration of the influence of age, sociologists, psychologists, and marketers did much to document the resistance to change common among the elderly [23]. The combined observations from the literature were best summarized with the recognition that the older the consumer, the more negative the view toward technology, and the lower the use of various technologies (including new prescription drugs).

While there was very little in the literature that considered the influence of gender on prescribing, there were also relatively few studies that were conducted in the area of customer (physicians) behavior on gender differences [24]. A review of the consumer behavior literature [25, 26], identified men as being more independent, confident, competitive, extremely motivated and more willing to take risks. The results of Inman and Pearce (1993) [27] suggested that female gender was strongly associated with the likelihood of not prescribing, or prescribing less than their male colleagues.

### Adoption and diffusion theory

Physician prescribing pattern is a very wide concept including various dimensions. In this research, the focus was on the adoption of drugs. The process of adoption often was referred to as the process of diffusion by which new ideas and products became adopted by society [28]. An undeniable fact is that marketing efforts have a significant impact on physicians’ decision to adopt [29] and can initiate the process of diffusion [30].

### Advertising theory

One of the main purposes of advertising was to entice the consumer to purchase the product [31], that was to prescribe as this is ultimately the metric against which pharmaceutical manufacturers measure success or failure.

Regardless of the role the physician occupied, in an environment in which the chooser is not the user, he is still the target of extensive pharmaceutical marketing within the context of advertising theory [32]. Therefore, the focus of this study was mainly on pharmaceutical marketing strategies as a factor influencing doctors’ decision. In their role as consumers, physicians created and store a set of preferred brands against which they simplify routine decision making.

In their role as consumers, physicians created and store a set of preferred brands against which they simplify routine decision making [33].

### Agency theory

Agency theory served this research well, as in an agency relationship, the principal delegates the decision-making authority to an agent to perform some action on the principal’s behalf [34].

In the context of the above definition, the manufacturer as principal depended on the physician as the agent to select drugs from their specific offering. The patient, in their role as principal, depended on the physician, acting as the agent, to choose or prescribe the appropriate drug. Saying that the theory of planned behavior [35] was primordial to understand physicians’ behavior as it relates to prescribing, and oft considering when attempting to modify or influence physician prescribing [36].

### Role theory

It served as a link between agency theory and the theory of planned behavior. It considered the foundations of the theory of planned behavior which are based upon an individual’s attitude toward the behavior, a perceived behavioral control and subjective norms [35] and agency theory, which focused on principal-agent relationships [37]. In this predefined context, role theory thus permitted better management/understanding of the dynamic aspects of the provider-client (agent-principal/physician-manufacturer) interface and centers on role performance and the interpersonal dimensions of service quality [38].

### Physicians as customers and relationship marketing theory

Pharmaceutical sales and pharmaceutical marketing analysts realized that the success of a brand depended mostly on the prescribing behavior (change to another brand) of the physician [39] who is the most crucial target customer for the pharmaceutical enterprises.

To remain profitable, monitoring the prescribing practice of each physician needed a suitable and successful relationship marketing program. The overall purpose of relationship marketing was to improve marketing productivity and enhance mutual value for the parties involved in the relationship. Consequently, instead of manipulating the customers (physicians) they were involved in the relationship [40].

As shown in Table 1 above, very few studies covered and studied the impact of pharmaceutical marketing strategies on the prescribing behavior of physicians in the developing countries of the middle-east. A report was published in “executive magazine” in 2015 by El-Jardali and Fadlallah [10] where it was found that 40% of all prescriptions in seven hospitals in Lebanon contained an error, of which 9% were unnecessary medication prescription.

**Table 1:** Summary of the studies that have addressed the impact of different pharmaceutical marketing strategies in developing middle-eastern countries

Authors	Site	Pharmaceutical marketing tool	Findings	Year
[7]	Yemen	Medical representatives	The majority of the physicians had positive interactions with medical representatives	2013
[51]	Tripoli, Benghazi and Sebha, Libya	Medical representatives	Cultivate subconscious commercial or conflict of interest relationships with prescribers	2013
[52]	Jordan	Gifts	There is a statistically significant effect of pharmaceutical companies’gifts on the doctors’prescribing behavior	2013
[53]	Baghdad, Iraq	Medical representatives, gifts, medical conferences and meetings	Acceptance of various types of gifts and it influences physician prescribing pattern and results in early adoption to prescribe newly medications	2014
[10]	Lebanon	Not defined	9% unnecessary medication prescription	2015
[41]	Lebanon	Pharmaceutical company representatives	Negatively impacting drug prescription and dispensing practices	2017
Not applicable	Not applicable	Not applicable yet	Not applicable yet	2017–2018

Source: Author’s elaboration based on data published in the literature review exploring the research objectives

The author set up a search alert on Google Scholar to be updated when new items that match his topic are published. In 2017, Hajjar, Bassatne, Cheaito, Naser El Dine, Traboulsy, Haddadin, Honein-AbouHaidar & Akl [41] studied the impact of only pharmaceutical representatives on drug prescription qualitatively and dispensing practices which were a negative impact. No later articles investigated other managing strategies in the Lebanese market regarding the topic of interest, leading to the particular need of this study. The next chapter formulated the hypothesis to be tested.

## Method

### Research philosophy

The epistemological orientation of this research was the positivism philosophy. The ontological orientation was objectivism. To test theories, we adopted a deductive research analysis method. We opted a quantitative method to measure the impact of pharmaceutical marketing strategies on the prescribing behavior of physicians and physicians’ opinion regarding ethical acceptability of gifts and samples along with any other characteristics that interested us. Therefore, in our cross-sectional study, we measured the impact of 10 pharmaceutical promotional tools across two age groups of physicians, under- 43 and over 43. We compared the impact of these tools among the two genders (male and female) and among physicians practicing in a hospital located in a Lebanese urban country and physicians practicing in another hospital located in a Lebanese rural country. The participants were asked to mention any additional promotional tool that they considered as a motivator for their prescription behavior.

### Data collection, study site, sampling method

Sampling was done by using a non-probability, quota, and convenience method. We chose this method because it is not feasible to draw a random probability-based sample of the population due to time considerations. The limitation of this method is that proportion of the entire population is not included in the sample group i.e. lack of representation of the entire population. Thus, the level of generalization of research findings compared to probability sampling is lower. The questionnaire format (Additional file 1) was written in english and was given to 364 Lebanese practicing physicians who are working in Lebanon at one of the two hospitals chosen by the author: a hospital in a Lebanese rural region and another one in a Lebanese urban region. However, 282 (response rate 77.47%) participated in the study and filled in the questionnaire completely. For this purpose, and to test the research hypotheses, a survey in a well- structured and self- administrated questionnaire format was developed and carried out by the researcher among the two hospitals of interest from March till April 2018. The questionnaire was piloted before final data collection to get at the thinking behind the answers so that we could accurately assess whether the questionnaire was filled out properly, whether respondents understood the questions, and whether the questions asked what we thought they were asking. Pre-testing also helped assess whether respondents were able and willing to provide the needed information. Twenty nine physicians undertook the pre-testing questionnaire, and their answers were excluded from the final analysis. No changes in the questions were recommended since the questions were clear and easy to understand. Thus, the link of the questionnaire was sent to each physician via “WhatsApp” after obtaining the approval from the Ethical Review Committee of each hospital. A reminder follow up notification was sent out after 7 days. The questionnaire included a covering letter questions to obtain demographic information about physicians, the influence of a list of 10 promotional tools used by most of the Lebanese pharmaceutical companies, questions regarding guidelines and physicians’ opinion regarding ethical acceptability of gifts and drug samples. Statistical Analysis was done using SPSS version 24 software for windows throughout the study to analyze the data.

Chi-square and Cramer’s V tests were carried out for the ten promotional tools identified through literature with the prescribing behavior of physicians that were respectively, shifting physicians’ drug prescription from one company to another if both drugs were generic and changing clinical practice after attending conferences and meetings.

Then the same test was applied to demographic parameters (gender and practice address) and physicians’ prescribing behavior. *P* values ≤0.05 were considered significant. Spearman’s Rho test was used to measure the strength of association between ordinal variables (age range, receiving of the Lebanese code of ethics and physicians’ prescribing behavior).

Doctors’ prescription behavior of drugs was taken as a dependent variable.

As independent variables, 10 main promotional tools used by pharmaceutical firms were taken into consideration, i.e. visits of medical representatives, sales calls made by pharmaceutical companies, drug samples, promotional drug brochures, medical equipment as gifts, low-cost gifts, sponsorship for travel or personal tour or expenses in conferences, direct mails, subscription of journals and participation by company in continuing medical education conferences. Also, physicians’ demographic parameters and their ethical opinion were considered as mediating variables. Reliability test and validating data were done using IBM SPSS statistics version 24 software, and the results showed that no rules were violated.

### Hypotheses development

As mentioned above, this study examined the impact of pharmaceutical marketing strategies on Lebanese physicians prescribing pattern concerning their demographic variables. Moreover, this study tested whether physicians consider gifts and samples acceptance as an ethical practice or an unethical one. For this purpose, the author addressed some questions that marketing managers of the drug companies are interested in: what is the most effective promotional tool in motivating Lebanese physicians to prescribe drugs? Do physicians’ demographic variables affect their perception towards different pharmaceutical marketing strategies? Do ethical codes affect physicians’ acceptance of gifts and samples? To answers these questions, this study formulated hypotheses based on theoretical background and summarized them in Table 2 below.

**Table 2:** Hypothesis to be tested in the Lebanese market

N° Hypothesis	Hypothesis
H 1	There’s a relationship between pharmaceutical marketing strategies and the prescribing behavior of physicians.
H 2	Accepting gifts by physicians is unethical.
H 3	There’s a relationship between physicians prescribing pattern and the location of practice.
H 4	There’s a relationship between physicians prescribing pattern and the age of physicians.
H 5	There’s a relationship between physicians prescribing pattern and the gender of physicians.

H1: Pharmaceutical marketing strategies change the prescribing behavior of physicians.

H2: Accepting gifts by physicians is unethical.

H3: Physicians with practices in rural settings are less likely to prescribe new drugs than are physicians operating in urban environments.

H4: Younger physicians are less likely to prescribe from new product categories.

H5: The probability of prescribing from a new drug category is greater among male than female physicians.

## Results

Two hundred eighty-two (response rate 77.47%) participated in the study and filled in the questionnaire completely. The majority of male practicing physicians liked to participate in the survey questionnaire (71.63%).

Results (Table 3) showed that 8 out of 10 dimensions taken as pharmaceutical marketing strategies were correlated to physicians prescribing behavior that is shifting drug prescription from one company to another if both were generic while seven dimensions out of 10 were correlated to this prescribing behavior that is changing clinical practice after attending conferences and meetings.

**Table 3:** The effect of 10 promotional tools on physicians’ prescribing patterns

	Professional’s prescribing behavior	
	Shifting drug prescription from one company to another if both were generic	Changing clinical practice after attending conferences and meetings
Pharmaceutical marketing strategies	Asymptotic significance (2-sided) *P* value	
Visits of medical representatives	.366	.149
Sales calls made by pharmaceutical companies	0.000	0.001
Drug samples	0.001	0.201
Promotional drug brochures	0.004	0.000
Medical equipment as gifts	0.004	0.078
Branded pen/ magnet/ mouse pad as gifts	0.004	0.011
Sponsorship for travel/ expenses in conferences/ sponsorship for personal tour	0.000	0.000
Direct mail	0.000	0.000
Subscription of journals	0.062	0.013
Participation by the company in continuing medical education conferences	0.000	0.000

Source: Survey

According to this study, the strategies used by pharmaceutical companies to promote their drugs demonstrated a correlation and a substantial relationship with physicians’ prescribing behavior. Therefore, the first hypothesis (there’s a relationship between pharmaceutical marketing strategies and the prescribing behavior of physicians) was accepted. Physicians declared that different promotional tools influenced them.

As shown in Table 4, the majority of these promotional tools were mostly or sometimes motivating physicians to prescribe promoted drugs. The two first tools that the majority of physicians agreed to be motivated mainly by were visits of medical representatives (34.8%) and drug samples (34.8%). Most physicians were sometimes influenced by promotional drug brochures (44.7%), medical equipment as gifts (41.5%). More than half of physicians (54.3%) agreed to be never influenced by sales calls made by pharmaceutical companies, and scarce were physicians who declared to be always affected by a particular pharmaceutical marketing strategy.

**Table 4:** Physicians’ evaluation of the motivational effect of each promotional tool on them to select and prescribe a certain product of a drug company

Parameter	1	2	3	4	5
Visits of medical representatives	46 (16.3%)	98 (34.8%)	83 (29.4%)	41 (14.5%)	14 (5%)
Sales calls made by pharmaceutical companies	0 (0.0%)	32 (11.3%)	46 (16.3%)	51 (18.1%)	153 (54.3%)
Drug samples	30 (10.6%)	98 (34.8%)	77 (27.3%)	47 (16.7%)	30 (10.6%)
Promotional drug brochures	10 (3.5%)	43 (15.2%)	126 (44.7%)	68 (24.1%)	35 (12.4%)
Medical equipment as gifts	0 (0.0%)	44 (15.6%)	117 (41.5%)	48 (17.0%)	73 (25.9%)
Branded pen/ magnet/ mouse pad as gifts	10 (3.5%)	10 (3.5%)	112 (39.7%)	76 (27.0%)	74 (26.2%)
Sponsorship for travel / expenses in conferences/ sponsorship for personal tour	32 (11.3%)	79 (28%)	68 (24.1%)	46 (16.3%)	57 (20.2%)
Direct mail	10 (3.5%)	15 (5.3%)	77 (27.3%)	95 (33.7%)	85 (30.1%)
Subscription of journals	0 (0.0%)	37 (13.1%)	98 (34.8%)	76 (27%)	71 (25.2%)
Participation by company in continuing medical education conferences	21 (7.4%)	89 (31.6%)	72 (25.5%)	73 (25.9%)	27 (9.6%)

1 = (always), 2 = (mostly), 3 = (sometimes), 4 = (rarely) 5 = (never)

Source: Survey

The results showed that pharmaceutical companies’ promotional tools moderately motivated physicians. Visits of medical representatives (34.8%), drug samples (34.8%), participation by the company in continuing medical education conferences (31.6%), sponsorship for travel/ expenses in conferences/ sponsorship for a personal tour (28%) could be considered as the most influential tools.

These results showed that practicing physicians didn’t add any new promotional tool other than those we suggested in the questionnaire. However, their answers revealed a need for more scientific evidence provided by continual medical education and by well-educated medical representatives. This scientific evidence will help them to make the decision and to prescribe the best drug among the others.

Moreover, results shown in Table 5 revealed that physicians considered gifts’ acceptance as a non-ethical practice and 53% of them used free samples offered by pharmaceutical companies to treat their patients. Therefore, the second hypothesis was rejected.

**Table 5:** The opinion of physicians regarding the acceptance of gifts

Questions	The answers			*P* value
	Yes/ always	Yes/ sometimes	No	
Do you think that low-cost gifts (pens, magnet, mousepad) for drug promotion from drug companies are acceptable?	34 (12.1%)	142 (50.4%)	106 (37.6%)	0.44
Do you think that the continuous supply of low-cost gifts to the physician at every visit of the medical representative is justifiable?	2 (0.7%)	102 (36.2%)	178 (63.1%)	0.847
Do you think that high-cost recreational gifts (like laptops, mobiles and LCD) are justifiable in drug promotion?	0 (0.0%)	71 (25.2%)	211 (74.8%)	0.175
Do you think that the continuous supply of high-cost gifts to the physician at every visit of the medical representative is justifiable?	0 (0.0%)	80 (28.4%)	202 (71.6%)	0.411

Source: Survey

H 3 was moderately accepted showing that there is a relationship between physicians prescribing pattern and the location of practice where changing in prescribing practices was more observable among physicians practicing in a hospital located in a rural region. H 4 was moderately accepted showing that there was a relationship between physician’s ages and prescribing pattern which is shifting generic drug prescription from one company to another. Regarding the last hypothesis, there was a relationship between physician’s gender and shifting drug prescription from one company to another, and H 5 was accepted (Table 6).

**Table 6:** Relationship between physicians’ prescribing behavior and demographic parameters

First parameter	Second parameter	*P* value
The location of practice	Shifting drug prescription from one company to another (if both drugs were generic)	0.001
	Changing clinical practice after attending conferences and meetings	0.023
Age	Shifting drug prescription from one company to another (if both drugs were generic)	0.000
	Changing clinical practice after attending conferences and meetings	0.121
Gender	Shifting drug prescription from one company to another (if both drugs were generic)	0.000
	Changing clinical practice after attending conferences and meetings	0.472

Source: Survey

## Discussion

Chi-square and Cramer’s v tests reveal that physicians’ prescribing behaviors are correlated with pharmaceutical promotional tools used in the Lebanese market. It was found that physicians are motivated by pharmaceutical companies’ promotional tools to prescribe promoted drugs. Similarly, some other studies found a direct correlation between physicians’ prescribing patterns and pharmaceutical promotional tools [20, 29, 30]. This can easily be explained by the fact that the persuasive effect of pharmaceutical marketing strategies put extra pressure on physicians to prescribe onerous, expensive drugs even when a cheaper generic drug would be appropriate [4].

This study found that physicians consider the following promotional tools as the most influential tools among the ten tools tested: visits of medical representatives, drug samples, participation by the company in continuing medical education conferences and sponsorship for traveling and personal tours. Thus, visits to medical representatives been perceived to be the most important and the most influential tool. Researchers suggest that physicians rely heavily on commercial sources of information such as detailer and that the more doctors rely on commercial sources of information, the less likely they are to prescribe drugs in a manner consistent with patient needs. Information provided by detailers is often biased and sometimes dangerously misleading [42, 43].

Regarding gift acceptance, the results of this study showed that there is no statistically significant difference among physicians who accept low and/or high-cost gifts and those who don’t. Moreover, statistically, there is no significance among physicians who consider the continuous supply of gifts (low and/or high-cost) at every visit of the medical representative as not justifiable and those who consider it as justifiable. However, the results showed that more physicians consider low cost-gift acceptance as an ethical practice than those who don’t (37.6%), but their continuous supply at every visit of the medical representative is more considered as not ethical (63.1%). This high percentage of physicians who do not accept the continuous supply of low-cost gifts means that some of the participated physicians in the study are not ashamed of the behavior of moderately accepting low-cost gifts. However, they know that gift acceptance is unethical, and they don’t accept it continuously. This could be explained by the fact that the Lebanese physicians are aware and are more considering the acceptance of small, low-cost gifts permissible than non-permissible even if the majority of them didn’t receive a copy of the 2016 code of ethics for medicinal products [19]. While in other studies, such as in Austria and Saudi Arabia, larger percent (66–80%) of physicians accept gifts from drug companies [44, 45].

For high-cost gifts, this study showed that most Lebanese physicians consider their acceptance as unethical (74.8%). The same is for the continuous supply of high cost-gifts (71.6%). While in Iraq and the United States of America, respectively 59 and 74.6% of physicians consider the acceptance of high-cost gifts inappropriate and unethical [44, 45]. Those high percentages can be explained by that physicians feel that small gifts do not significantly alter or influence their prescribing pattern but expensive gifts might do so [46]. Again, these results may give us a hope in that physicians are conscious of unethical expensive gifts’ acceptance.

However, social scientists demonstrated that the tendency to reciprocate for gifts, even the small ones, is a powerful influence on people’s behavior. Individuals who receive gifts are often unable to remain objective. Whenever a physician accepts an award, an implicit relationship is established between the doctor and the company resulting in a prescription [3]. Physicians not acknowledging the power of receiving small gifts are more likely to be influenced because their defenses are down.

Regarding free samples, it was found in this study that most physicians use free samples to treat their patients (53%). These free samples could be provided for a poor patient who cannot afford to buy it and therefore who manages drug costs in the long term or in some cases to initiate treatment for a new patient.

This study showed that Lebanese physicians significantly shift drug prescription from one company to another if both drugs were generic and they change their clinical practice. This means that the prescribing behavior of Lebanese physicians is highly influenced by pharmaceutical marketing strategies. Similarly, there are some other studies where it was showed that physicians who are interested in drug promotion and who have a positive attitude toward pharmaceutical companies’ promotions adopt rapidly prescription sponsored medications [47]. From another hand, previous studies showed that most physicians don’t feel that drug companies influence their prescribing pattern [48]. The difference in this study may result from the indirect way of questioning, which may provide a more realistic result than other studies.

It was found that the perception of practicing physicians towards some promotional tools’ influence on their prescribing behavior is dependent on demographic factors that are practice address, age, and gender of physicians. Practicing physicians in the hospital located in the urban region assign a greater shift in prescribing drugs as compared to physicians practicing in the hospital located in the rural area. Usually, physicians practicing in the rural areas do not get high access to new information unlike physicians practicing in urban regions; this may be one of the reasons for assigning more significant influence to promotional tools by physicians practicing in the urban area. These findings are in accordance with the findings in previous researches by Tamblyn, McLeod, Hanley, Girard, and Hurley (2003) [22] and Cutts and Tett (2003) [49].

This empirical investigation confirmed that older physicians assign to be less influenced by pharmaceutical promotional tools as compared to younger physicians. As older the consumers (physicians) are, the more the view is negative towards technology, and the lower the use of various technologies (including the prescription of new drugs). In other words, older physicians are less modern than younger ones. These results are similar to the results found by Peay and Peay’s work in 1994 [50]. They suggested that older doctors were less innovative than younger ones.

Moreover, it was found in this study that the probability of prescribing from a new drug category (prescribing behavior) is greater among female than male physicians.

The general questions which were asked to practicing physicians to get more insights regarding the improvement of regulations framing the relationship between physicians and pharmaceutical companies confirm the previous results obtained in this study. The answers showed that physicians investigated are conscious and aware of the need for more strengthening the ethical norms regulating their interaction with pharmaceutical industries. This interaction should be with medical representatives holding a certificate of professional and ethical capability to execute a given profession, and the number of visits should be regulated. Besides, physicians appreciate that pharmaceutical companies invite them to international congresses which means that they are interested in acquiring scientific knowledge.

### Contributions of this research (theoretical, managerial)

This present study adds to the previous researches that conceptualize the fact that clinical practice decision making is a dynamic process which is affected by some pharmaceutical marketing strategies. Although the doctors generally use the pharmacological criteria in deciding which drug to prescribe, the findings of the study show that the demographic influences are also rated essential factors in the doctor’s decision to prescribe. The present study identified and analyzed the demographic variables like the gender of the doctors, the age of doctors and practice location of the doctors and the results reveal that they have an impact on prescribing behavior.

Our empirical investigation of physicians’ prescription behavior in Lebanon and its findings contribute to an improvement in the marketing practices of the pharmaceutical industry. Knowing that when prescribing habits are once learned, it may be difficult to change them, pharmaceutical managers should use appropriate promotional tools and target the category of physicians that are more influenced than the others. Therefore, the pharmaceutical company optimizes its marketing expenditures better than competitors and increases its sales with lower promotional budgets.

Therefore, the results imply that public policymakers should take prescription behavior seriously and conduct more prescription behavior studies at regular intervals, to precisely understand the impact of tangible rewards on physicians’ prescribing patterns and control unethical practices by both pharmaceutical companies and physicians. So, it is recommended to establish guidelines and to be sure that all physicians are aware of them and always to keep physicians updated regarding new guidelines and to control guidelines’ implementation to limit unethical promotion practices and unethical prescription patterns.

These results, although not surprising, add to the existing body of information because they come from direct observations of behavior in a non-probability, quota and convenience sample.

### Limitations and suggestions for future research

Although this study is limited by maturation that is quite simply, people change, and the ways in which they change may have implications for the dependent variable, but it rings a bell of danger by the non-ethical interaction between practicing physicians and drug companies in Lebanon, which negatively affect physicians prescribing behavior and in turn affect patients’ health negatively. It is suggested for future research to replicate this study and include pharmaceutical companies and pharmacists since they play a role in the pharmaceutical market. Also, the same research can be replicated by including more demographic factors of the physicians.

## Conclusions

Most of the investigated physicians change their prescribing behavior, and it can simply be concluded that prescribing pattern of Lebanese physicians is negatively affected by promotion tactics.

It can be concluded that the pharmaceutical marketers have to understand the real needs, beliefs, and behaviors of physicians towards their marketing and promotional tools while taking into account physicians’ demographic factors and physicians’ opinion regarding ethical acceptability of gifts and samples. Physicians are the most substantial determinants in pharmaceutical sales by deciding which drug will be used by patients. Influencing the physician is a key to pharmaceutical sales. The marketing efforts of drug companies must target female, young physicians practicing in rural regions. This might generally be the most influenced category of practicing physicians.

## Additional file

Additional file 1: Questionnaire format. (DOCX 220 kb)
