# Supplementary figures and images for: Pharmaceutical marketing strategies’ influence on physicians' prescribing pattern in Lebanon: ethics, gifts, and samples
Source: BMC Health Serv Res. 2019 Jan 30;19:80. doi: 10.1186/s12913-019-3887-6 (PMC6354386; doi:10.1186/s12913-019-3887-6)

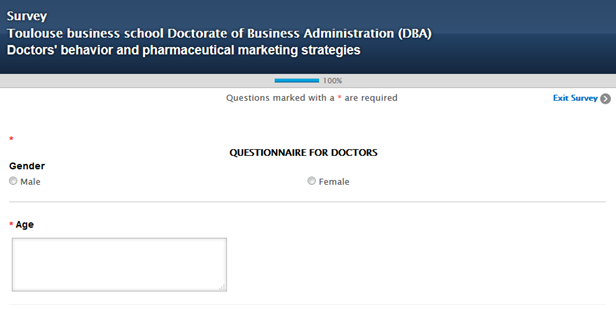


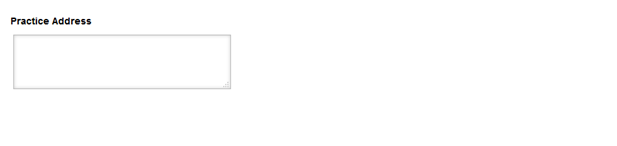

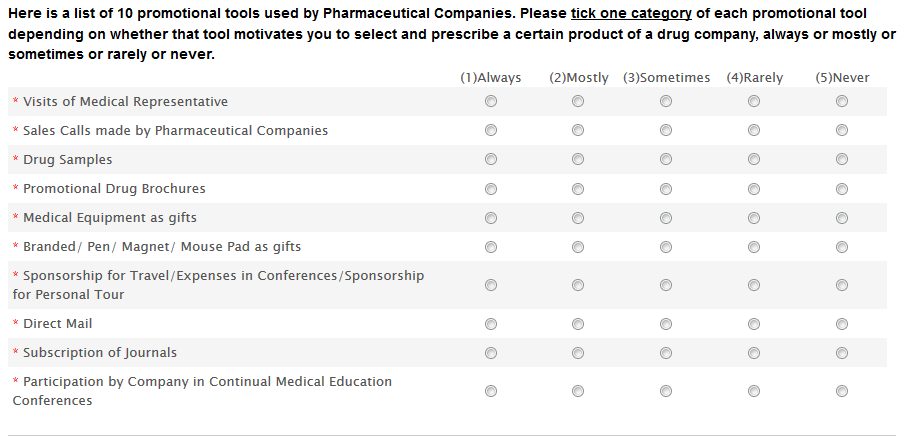


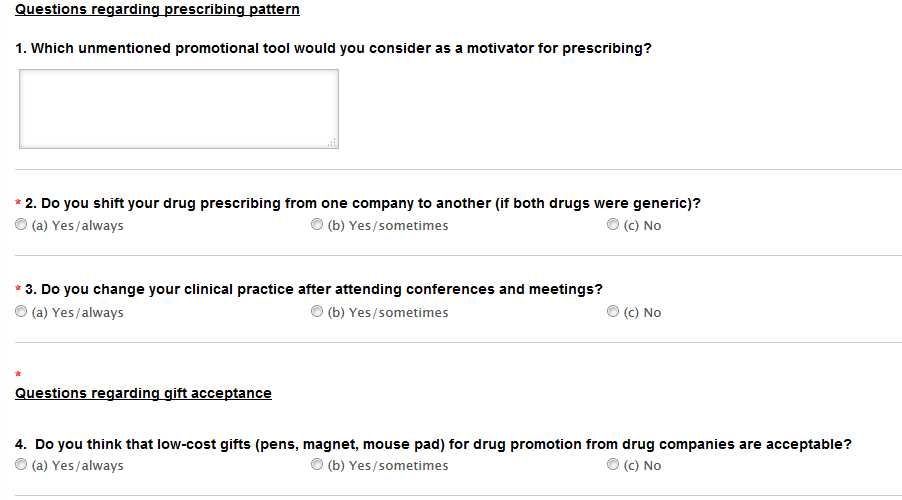


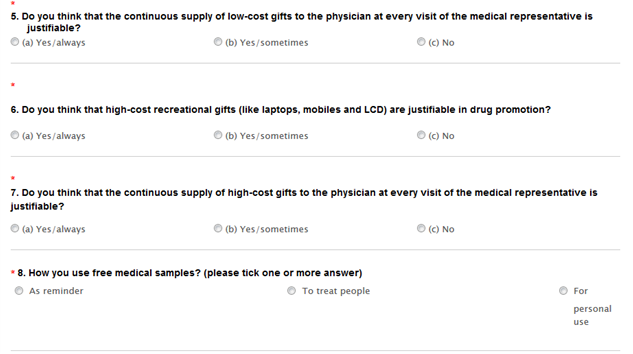


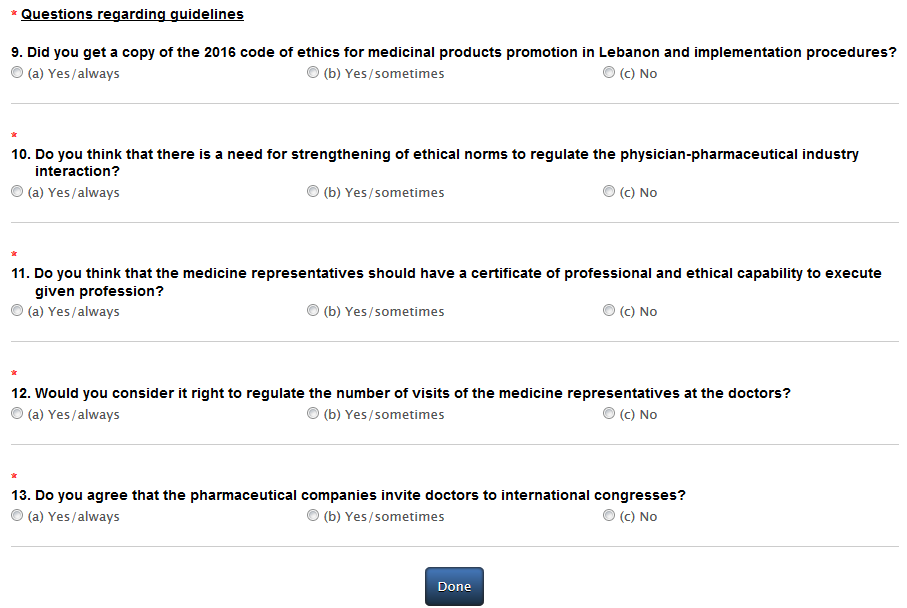

Supplement: Supplementary file 1 — Questionnaire format. (DOCX 220 kb) [file 12913_2019_3887_MOESM1_ESM.docx]
